# Association between *IL23R* and *ERAP1* polymorphisms and sacroiliac or spinal MRI inflammation in spondyloarthritis: DESIR cohort data

**DOI:** 10.1186/s13075-018-1807-5

**Published:** 2019-01-15

**Authors:** Adeline Ruyssen-Witrand, Cécile Luxembourger, Alain Cantagrel, Delphine Nigon, Pascal Claudepierre, Yannick Degboe, Arnaud Constantin

**Affiliations:** 1 Centre de rhumatologie, CHU de Toulouse, UMR 1027, Inserm, Université Paul Sabatier Toulouse III, Toulouse, France; 2 Centre de rhumatologie, CHU de Toulouse, UMR 1043, CPTP, Inserm, Université Paul Sabatier Toulouse III, Toulouse, France; 3 Centre de rhumatologie, CHU de Toulouse, Toulouse, France; 4 Departement de Rhumatologie, Henri Mondor Hospital, APHP, Université Paris Est Créteil, EA 7379 – EpidermE, F-94010 Créteil, France

**Keywords:** Spondyloarthritis, Sacroiliitis, Polymorphisms

## Abstract

**Background:**

To investigate the association between 12 single nucleotide polymorphisms (SNPs) located on *ERAP1* and *IL23R* with the presence of inflammation on the sacroiliac joint (SIJ) or spinal magnetic resonance imagery (MRI) in an early onset spondyloarthritis (SpA) cohort.

**Methods:**

All the patients included in the DESIR cohort with an axial SpA and available DNA at baseline were enrolled in this study (*n* = 645 patients) and underwent a clinical examination, CRP assay, SIJ and spinal MRI scans. Six SNPs located on *ERAP1* (*rs30187*, *rs27044*, *rs27434*, *rs17482078*, *rs10050860*, *rs2287987*) and six SNPs located on *IL23R* (*rs1004819*, *rs10489629*, *rs1343151*, *rs2201841*, *rs10889677*, *rs11209032*) were genotyped. Univariable analyses were performed to test the association between the genotypes and SIJ and spinal MRI inflammation, as well as disease activity based on Bath Ankylosing Spondylitis Disease Activity Index (BASDAI), Ankylosing Spondylitis Disease Activity Score-C-Reactive Protein (ASDAS-CRP) and CRP.

**Results:**

One SNP located on *ERAP1* (rs27434) and haplotype CCT of *ERAP1* were associated with SIJ inflammation detected by MRI, but these associations were below the Bonferroni corrected threshold of significance. However, one SNP (rs1004819) located on *IL23R* was associated with SIJ MRI inflammation (rs1004819: TT 42.3%, CT 40.5%, CC 26.5%, *p* = 0.0005). This locus was also significantly associated with Spondyloarthritis Research Consortium of Canada scores while no association with another inflammatory parameter such as BASDAI, ASDAS-CRP, CRP or Berlin MRI score was identified in this population.

**Conclusion:**

One locus of the *IL23R* gene was associated with SIJ MRI inflammation and might be a marker of more active disease in recent onset SpA.

**Trial registration:**

clinicaltrials.gov, NCTO 164 8907

## Background

Spondyloarthritis (SpA) is a common, highly heritable form of inflammatory arthritis that primarily affects the spine and pelvis. The first genetic determinant of this disease is the presence of *HLA-B27* susceptibility alleles for which 114 subtypes are now recognised [1].

The presence of *HLA-B27* is the major genetic factor implicated in disease susceptibility. Nevertheless, other genes are also involved in the genetic background for SpA since *HLA-B27* is detected in around 90% of patients with ankylosing spondylitis (AS) and in 30–50% of cases involving other SpA subtypes such as psoriatic arthritis (PsA) or SpA associated with inflammatory bowel disease [2].

Several genome-wide association (GWA) studies have been published on AS susceptibility in individuals from European and Asian populations, which have identified at least 31 significant non-HLA genetic loci. These genes account for up to 25% of the overall heritability of AS, with the overwhelming majority provided by *HLA-B27* per se [2, 3]. Among these recently identified loci, variations within genes encoding proteins involved in interferon or tumour necrosis factor/NFkB signalling and transcription have been identified, as well as genes influencing IL-23/IL-17 signalling or TCD8+ differentiation or genes encoding proteins crucial for antigen presentation [3].

Among these novel loci, *ERAP1* variants have been widely replicated by GWA studies in populations of white European ancestry and in Eastern Asians [4, 5]. *ERAP1* variants alter the quantity, length and stability of peptides presented to the peptide-MHC molecule [6]. Furthermore, several variants located on *IL23R* have also been shown to be associated with SpA susceptibility in several studies [3, 7, 8].

Although these associations have often been replicated in several different populations, the association of these polymorphisms with the disease phenotype and especially disease activity assessed by magnetic resonance imaging (MRI) inflammation has been poorly investigated.

The aim of this study was to investigate the association between 12 SNPs located on *ERAP1* and I*L23R* genes and sacroiliac joint (SIJ) or spinal MRI inflammation in a cohort of newly diagnosed SpA patients (DESIR cohort).

## Patients and methods

### Study population

The DEvenir des Spondyloarthrites Indifférenciées Récentes (DESIR) cohort has already been described in the literature [9]. In short, DESIR is a French, long-term prospective longitudinal cohort study monitoring 708 patients (between 18 and 50 years of age) presenting inflammatory back pain (IBP) suggestive of axial SpA for ≥ 3 months and < 3 years, according to their rheumatologist, included in 25 centres across France between 2007 and 2010. All patients included in the study underwent a baseline collection of clinical data, blood samples, unenhanced pelvic and spinal X-rays, SIJ and spinal MRI. Among these patients, 645 with DNA specimens and fulfilling at least 1 spondyloarthritis classification criteria including axial Assessment of Spondyloarthritis international Society (ASAS) criteria 2009 [10], Amor criteria [11], ESSG criteria [12] or modified New York criteria [13] were selected for the present study. The baseline database of the DESIR cohort was used for this study (dataset locked on June 30, 2010).

### Ethics, consent and permission

The study complied with Good Clinical Practice Guidelines (clinicaltrials.gov: NCTO 164 8907, https://clinicaltrials.gov/ct2/show/NCT01648907) and was approved by the appropriate Medical Ethics Committee (CPP Ile-de-France III, submission number P070302). Patients gave their informed consent before the study started, including their permission for any anonymously processed data to be published.

### Genetic polymorphisms

Genotyping of the *IL23R* (*rs1004819*, *rs10489629*, *rs1343151*, *rs2201841*, *rs10889677*, *rs11209032*) and *ERAP1* (*rs30187*, *rs27044*, *rs27434*, *rs17482078*, *rs10050860*, *rs2287987*) polymorphisms was performed by LGC Genomics Platform (UK) using allele-specific kinetic polymerase chain reaction analysis and the KASPar method (accuracy generally > 99%, error rate < 0.3%). These 12 SNPs were selected because they were associated with SpA susceptibility [3–8] or located close to a SNP associated with SpA susceptibility with an allele frequency > 0.1 in the Caucasian population.

### Imaging data

An MRI scan of the SIJ and spine was performed at the outset in each participating centre using 1.0–1.5 T machines.

### MRI sacroiliitis definition as primary outcome

The acquired sequences were coronal oblique T1-weighted FSE and STIR images with 12–15 semi-coronal slices, each 4-mm-thick, parallel to the long axis of the sacrum. All available baseline SIJ MRIs were read centrally and independently by 2 readers, blinded for all clinical and laboratory data. SIJ MRI scans were considered positive according to the ASAS definition [14] if bone marrow oedema lesions, highly indicative of SpA, were present (i.e. if ≥ 1 bone marrow oedema lesions were visible on ≥ 2 consecutive slices, or if several bone marrow oedema lesions were visible on a single slice). Inter-reader agreement for the definition of MRI-detected sacroiliitis was good (kappa score of approximately 0.74) [15]. When central readers disagreed on the presence of inflammation on the spine or SI MRI, a senior radiologist involved the MRI and served as adjudicator blinded to the information of the primary readers.

### Other secondary imaging outcomes

The two readers also scored the MRI scans (intra-class correlation coefficient of around 0.94 for absolute baseline scores) for the presence of inflammatory lesions according to the Spondyloarthritis Research Consortium of Canada (SPARCC) scoring method (range 0–72) [16].

MRI scans of the thoracic spine and lumbar spine were performed using the T1-weighted spin-echo and short-tau inversion recovery sequences. Inflammatory lesions were centrally scored by two independent readers, blinded to the clinical data. A positive spinal MRI was defined according to the ASAS consensus (i.e. ≥ 3 inflammatory lesions) [17] and using the Berlin method [18]. Agreement between readers for an ASAS-defined positive spinal MRI was moderate (*ƙ* = 0.58) [19].

All MRI scans were centrally scored by two trained readers and expressed in positive MRI sacroiliitis according to the ASAS definition or not, size of bone marrow oedema according to the Spondyloarthritis Research Consortium of Canada (SPARCC) scoring method (range 0–72) and positive spinal MRI according to the ASAS consensus definition and MRI spine inflammation using the Berlin method.

Sacroiliitis on X-rays was centrally assessed according to the modified New York criteria [13] by two different, blinded, trained readers (moderate inter-reader agreement with a kappa score of around 0.54) [20].

### Other clinical and biological data

For the present study, demographic characteristics including age, sex, disease duration and disease activity data were analysed at the outset. The markers of disease activity included the Bath Ankylosing Spondylitis Disease Activity Index (BASDAI) and the Ankylosing Spondylitis Disease Activity Score-C-Reactive Protein (ASDAS-CRP) and were studied as potential cofounders for the primary analysis.

C-reactive protein (CRP) and *HLA-B27* status were measured at baseline and studied as potential cofounders for the primary analysis.

### Statistical analysis

Tests for deviation from the Hardy-Weinberg equilibrium (HWE) were performed using a standard *χ*^2^ test (1 d.f.). A linkage disequilibrium was investigated within SNPs located on the same gene using PLINK v1.07 software, and in the case of a strong linkage disequilibrium (defined by a *r*^2^ > 0.8 and *D*′ = 1), a haplotype of the SNPs in linkage disequilibrium was created for analytic purposes with Haploview software.

The primary outcome was to investigate the association between the SNPs and haplotypes of SNPs and SIJ MRI inflammation using a Fisher test or chi-square test and the Cochrane-Armitage test for trends. A Bonferroni correction was applied to the result of the Cochrane-Armitage trend test for this analysis taking multiple tests into account. In case of association, epistatic interactions between the locus and *HLA-B27* were investigated using two different methods: the RERI method and PLINK epistasis software v1.07 [21]. In the event of an interaction, the results were stratified on *HLA-B27* carriage.

If a significant association was detected, other associations between the SNPs and/or haplotypes and activity assessed by BASDAI, ASDAS-CRP, CRP, MRI oedema size assessed by using the SPARCC score or structural damage radiographic sacroiliitis (New York criteria) were further assessed, using a Fisher test or chi-square test and the Cochrane-Armitage test for trends in the case of qualitative variables or Kruskal-Wallis and Cuzick trend tests for quantitative variables. Considering all the multiple tests performed, the threshold of significance after Bonferroni correction was set at approximately 0.0033. Assuming that a meaningful difference of 15% in the prevalence of MRI sacroiliitis between allele carriers and non-carriers, and knowing that 34% of the DESIR patients had a MRI sacroiliitis, and that minor allele prevalence of the different SNPs was about 0.3 in most cases, the power to identify an association with an alpha risk of 0.0033 was estimated between 74 and 80%.

Finally, to investigate whether the association between the SNPs were independently associated with MRI sacroiliitis, a multivariable analysis using logistic regression, backward procedure, was performed including other potential cofounding variables such as age, gender, *HLA-B27* presence, CRP, ASDAS-CRP and NSAIDs intake.

## Results

All of the 12 SNPs fit the Hardy-Weinberg equilibrium expectations (*p* > 0.05) and had an allele frequency > 0.10 in the DESIR cohort. The genotyping success rate was approximately 98.8%, and 645 patients could be enrolled in this study whose characteristics are summarised in Table 1.

**Table 1:** Baseline characteristics of the patients enrolled

	Study population, *n* = 645
Age, med (IQR)	32.9 (26.5–39.7)
Gender, number of males (%)	299 (46.4)
Disease duration, months, med (IQR)	17.1 (9.6–26.5)
Positive *HLA-B27*, number (%)	403 (62.6)
BASDAI, med (IQR)	46 (29–61)
ASDAS-CRP, med (IQR)	2.7 (2.0–3.4)
CRP (mg/L), med (IQR)	3.2 (2–8)
NSAIDs intake, *n* (%)	451 (71.5)
Radiographic sacroiliitis, *n* (%)	184 (28.9)
mSASSS, med (IQR)	0 (0–0)
Presence of MRI sacroiliitis, *n* (%)	214 (34.2)
MRI SPARCC, med (IQR)	0 (0–4)
ASAS positive spinal MRI, *n* (%)	49/603 (8.1)
Spinal MRI Berlin score, med (IQR)	0 (0–0)

*BASDAI* Bath Ankylosing Spondylitis Disease Activity Index, *ASDAS-CRP* Ankylosing Spondylitis Disease Activity Score with CRP, *mSASSS* Modified Stoke Ankylosing Spondylitis Spine Score, *SPARCC* Spondyloarthritis Research Consortium of Canada scoring system (sacro-iliac joints MRI scoring system), *Berlin score* spinal MRI scoring system

Three SNPs located on the *ERAP1* gene (rs17482078, rs10050860, rs2287987) demonstrated strong linkage disequilibrium (*r*^2^ ranging between 0.97 and 0.99, *D*′ = 1), and a haplotype of these three loci was thus created and used for further analyses. Two SNPs located on the *IL23R* gene (rs2201841 and rs10889677) also showed strong linkage disequilibrium (*r*^2 2^ = 0.96, *D*′ = 1), and a haplotype including these two loci was created and used for further analyses.

### Association between *ERAP1*, *IL23R* and SIJ MRI inflammation

None of the three SNPs and the haplotype located on *ERAP1* was associated with SIJ MRI inflammation when applying Bonferroni correction.

**Fig. 1:**
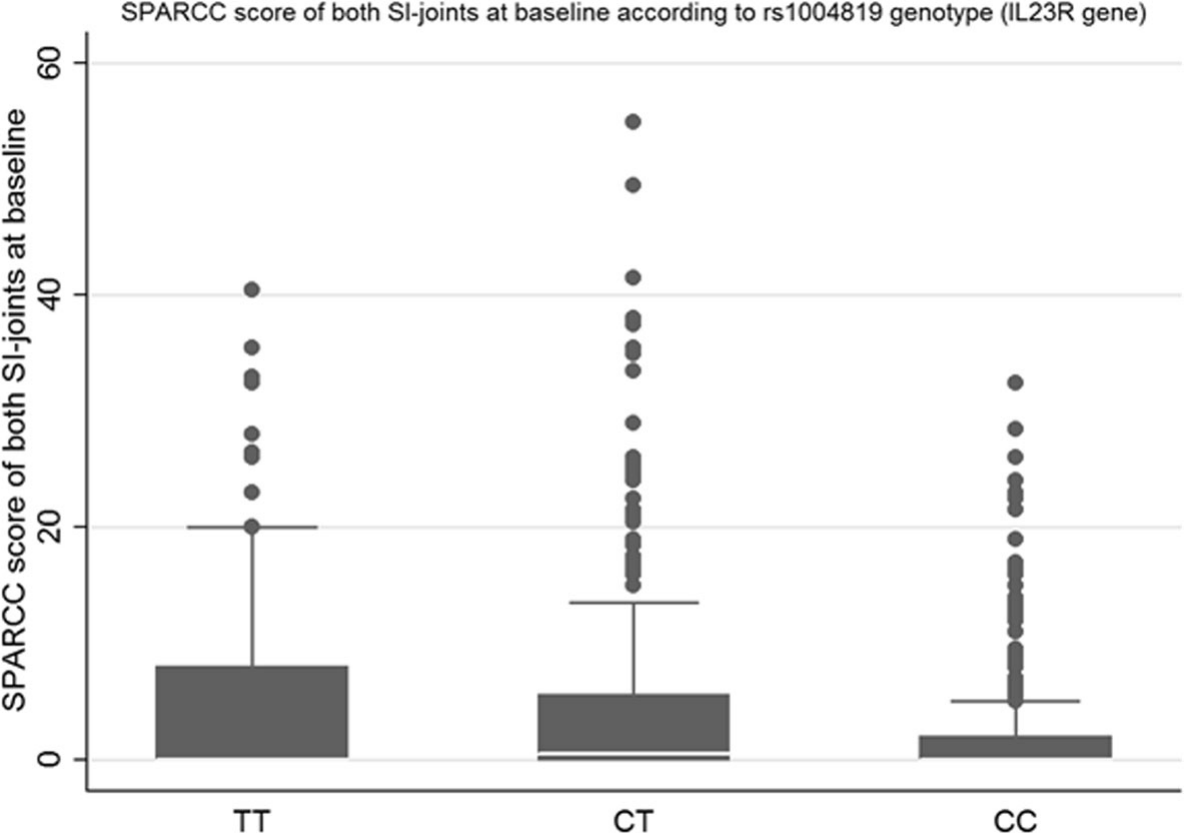
SPARCC scores of sacroiliac joint MRI according to the genotypes of rs1004819 and GA haplotype of *IL23R*. SPARCC, Spondyloarthritis Research Consortium of Canada scoring system (sacroiliac joints MRI scoring system)

After applying a Bonferroni correction, only the rs1004819, located on *IL23R*, remained significantly associated with the presence of SIJ MRI inflammation and was thus further investigated (Table 2). This locus was thus also associated with sacroiliac oedema size assessed by SPARCC score (*p* for the association, 0.001; Table 3, Fig. 1). However, this locus was neither associated with other inflammatory parameters (ASDAS CRP, BASDAI, CRP of MRI spinal inflammation assessed by Berlin score) nor radiographic sacroiliitis (Table 3).

**Table 2:** Association between MRI sacroiliac inflammation and *IL23R* and *ERAP1* SNP and haplotype genotypes

	Number of patients (%)	*p* value comparison of distribution	*p* value for Cochran-Armitage trend test
ERAP1			
rs27044			
GG	21 (39.62)	0.5	0.25
CG	98 (35.25)		
CC	94 (32.19)		
rs30187			
TT	36 (37.5)	0.07	0.04
CT	113 (37.5)		
CC	65 (28.4)		
rs27434			
GG	105 (30.3)	0.04	0.01
AG	92 (38.1)		
AA	16 (47.1)		
Haplotype CCT*			
0 copy	4 (14.8)	0.02	0.007
1 copy	55 (29.4)		
2 copies	153 (37.2)		
IL23R			
rs1004819			
TT	30 (42.3)	*0.001*	*0.0005*
CT	109 (40.5)		
CC	75 (26.5)		
rs10489629			
GG	35 (29.2)	0.3	0.1
AG	101 (33.8)		
AA	77 (38.1)		
rs1343151			
TT	24 (30.4)	0.02	0.02
CT	82 (29.5)		
CC	108 (40.3)		
rs11209032			
GG	74 (35.4)	0.03	0.01
AG	109 (47.3)		
AA	26 (41.3)		
Haplotype GA*			
0 copy	80 (28.9)	0.02	0.0054
1 copy	103 (37.6)		
2 copies	30 (44.1)		

*: Haplotype CCT: rs17482078, rs10050860, rs2287987; haplotype GA: rs2201841, rs10889677

**Table 3:** Association between rs1004819 located on *IL23R* genotypes and clinical and biological activity, SPARCC score and MRI-detected spinal inflammation

rs1004819, by genotype	TT	CT	CC	Comparison* across genotypes *p* value
BASDAI, med (IQR)	38 (24–53)	48 (30–62)	48 (28–62)	0.1
ASDAS-CRP med (IQR)	2.5 (1.7–3.3)	2.8 (2.0–3.4)	2.7 (2.0–3.4)	0.5
CRP, med (IQR)	3.6 (2.0–8.0)	3.5 (2.0–7.0)	3 (2.0–8.8)	0.3
SPARCC, med (IQR)	0.0 (0.0–8.0)	0.5 (0.0–5.5)	0.0 (0.0–2.0)	*0.0008*
Patients with MRI spinal inflammation, *n* (%)	11 (15.9)	20 (7.8)	18 (6.6)	0.03
Berlin score, med (IQR)	0 (0–1)	0 (0–0)	0 (0–0)	0.09

*p value with a Cochrane-Armitage trend test in case of dichotomous variable and Cuzick trend tests in case of continuous variable

*BASDAI* Bath Ankylosing Spondylitis Disease Activity Index, *ASDAS-CRP* Ankylosing Spondylitis Disease Activity Score with CRP, *mSASSS* Modified Stoke Ankylosing Spondylitis Spine Score, *SPARCC* Spondyloarthritis Research Consortium of Canada scoring system (sacroiliac joints MRI scoring system), *Berlin score* spinal MRI scoring system

### Epistatic interaction between rs1004819 on *IL23R* and *HLA-B27* allele carriage

Epistatic interactions between the SNP of IL23R and *HLA-B27* were investigated to assess whether *HLA-B27* allele carriage could interact with the association between rs1004819 and MRI sacroiliitis using two different methods: the RERI method and PLINK epistasis software v1.07 [21]. No epistatic interaction was identified between *rs1004819* and *HLA-B27* allele carriage for the association with MRI sacroiliitis.

### Mutivariable analysis assessing the risk of MRI sacroiliitis according to rs1004819 genotype

After logistic regression including other cofounding variables such as gender, age, *HLA-B27* presence, CRP, ASDAS-CRP and NSAIDs intake, rs1004819 genotype remained independently associated with MRI sacroiliitis (Table 4).

**Table 4:** Logistic regression assessing the risk of MRI sacroiliitis and including rs1004819 genotype, age, gender, *HLA-B27* presence and NSAIDs intake

	OR [95%CI]	*p* value
rs1004819	0.71 [0.53–0.94]	0.02
age	–	NS
Gender	2.15 [1.46–3.19]	< 0.0001
CRP (per mg/L)	1.02 [1.00–1.03]	0.01
ASDAS-CRP	–	NS
*HLA-B27*	1.95 [1.27–2.97]	0.002
NSAIDs intake	–	NS

Age, ASDAS-CRP and NSAIDs intake were removed after backward procedure

*OR* odds ratio, *%95CI* 95% confidence interval, *NS* non-significant

## Discussion

This work aimed to investigate the association between 12 loci located on *ERAP1* and *IL23R* genes and SIJ or spinal MRI inflammation in a cohort of 625 patients with recent onset axial SpA (DESIR cohort). In this study, rs1004819 located on the *IL23R* gene was associated with the presence of MRI sacroiliitis and MRI bone oedema size assessed by the SPARCC score. However, this locus was not associated with the clinical and biological activity, MRI spinal inflammation nor with radiographic sacroiliitis. None of the SNPs studied on ERAP1 was significantly associated with SIJ or spinal MRI inflammation in this study.

IL-23 is a pro-inflammatory cytokine formed via the binding of a shared IL-12p40 unit to a p19 protein secreted primarily by dendritic cells, monocytes and macrophages [22]; IL-23 plays a role in the maintenance of immune responses by controlling T cell memory function and by activating the proliferation and survival of Th17 cells. IL-23 is thus a key cytokine in several inflammatory diseases including SpA [23]. The *IL23R* gene is located on chromosome 1 (1p31). In recent years, new therapeutic biological agents targeting the IL-17/IL-23 pathway have proved effective in reducing disease activity, and studies have suggested a preventive role in the structural radiographic progression of spondyloarthritis and psoriatic arthritis [24, 25]. ERAP1 molecules encoded in the endoplasmic reticulum lumen play an important role in peptide trimming for presentation at the cell surface. Reduced trimming of peptides and consequently altered antigen presentation on HLA-B27 molecules is probably an important mechanism involved in AS development and protection [26].

Several studies have previously shown an association between rs11209026 and AS susceptibility [3], as well as radiographic sacroiliitis [27]. In our study, this locus was not genotyped because the allele frequency of this variant is very low (A allele frequency approximately 0.04 in the general population), and as the study involved a small sample, the power for identifying an association was allegedly too low to be conclusive.

No significant epistatic interaction was identified between *rs1004819* on *IL23R* and the presence of *HLA-B27*, thus indicating that the *IL23R* locus is associated with MRI inflammation independently of *HLA-B27* allele carriage.

This study has several limitations. Firstly, the work was set on a sample of 625 patients and multiple loci were tested; thus, the power to detect a difference was decreased. However, power calculation in SNPs where minor allele prevalence was about 0.3 revealed a power to detect a difference about 15% between 74 and 80%. Furthermore, we did not have a second sample to replicate our results, and these findings have to be further confirmed in an independent population. However, this locus is already known to be associated with AS susceptibility and it is established that MRI sacroiliitis can be considered as an early stage of radiographic sacroiliitis and thus predispose to AS disease. In this study, we chose to apply a candidate gene search strategy to identify association between loci already known as susceptibility markers of the disease and MRI inflammation in early SpA patients. Other strategies such as *IL23R* and *ERAP1* DNA sequencing could also be performed to further investigate the association between these genes and MRI inflammation in SpA. Finally, this study investigated only SpA patients with no control group. Thus, these findings identified associations between candidate genes and different phenotypes of SpA, and this study did not demonstrate here that these loci were SpA susceptibility markers.

To the best of our knowledge, this is the first study highlighting the association between genetic polymorphisms outside of the MHC region known to be associated with AS susceptibility and MRI inflammation in recent onset axial SpA patients. DESIR cohort imaging data was centrally scored by experienced readers blinded to the clinical data, which improves imaging data confidence. The rs1004819, located on *IL23R*, that is associated with MRI sacroiliitis, has proved to be strongly associated with disease susceptibility. It has also proved to be an apparent marker of inflammatory disease in patients diagnosed with recent onset axial SpA, since it was also associated with MRI sacroiliitis oedema size, assessed via the SPARCC scoring system, and with the presence of an MRI spinal inflammatory lesion.

## Conclusion

In summary, this study identified an association between one locus on the *IL23R* gene and MRI inflammation located on the spine and SIJ. However, these findings should be replicated in other populations and the role played by these polymorphisms in *IL23R* gene expression should be further explored.

## Abbreviations

AS: Ankylosing spondylitis
ASAS: Assessment of Spondyloarthritis international Society
ASDAS: Ankylosing Spondylitis Disease Activity Score
axSpA: Axial spondyloarthritis
BASDAI: Bath Ankylosing Spondylitis Disease Activity Index
BASFI: Bath Ankylosing Spondylitis Functional Index
bDMARD: Biologic disease-modifying antirheumatic drugs
CRP: C-reactive protein
DESIR: DEvenir des Spondyloarthites Indifférenciées Récentes
ESSG: European Spondyloarthropathy Study Group
GWA: Genome-wide association
HLA: Human leucocyte antigen
HWE: Hardy-Weinberg equilibrium
IBD: Inflammatory bowel disease
MHC: Major histocompatibility complex
MRI: Magnetic resonance imaging
NSAID: Non-steroidal anti-inflammatory drug
PsA: Psoriatic arthritis
SD: Standard deviation
SIJ: Sacroiliac joints
SNP: Single nucleotide polymorphism
SpA: Spondyloarthritis
SPARCC: Spondyloarthritis Research Consortium of Canada
TNF: Tumour necrosis factor
VAS: Visual analogue scale
